# Physicochemical Stability of an S-Ketamine-Dexmedetomidine Anesthetic Mixture: An In Vitro Study

**DOI:** 10.7759/cureus.112087

**Published:** 2026-07-05

**Authors:** Sergio Zuazo, Bruno Santiago

**Affiliations:** 1 Anesthesiology, University of São Paulo, São Paulo, BRA; 2 Anesthesia, State University of Rio de Janeiro, Rio de Janeiro, BRA

**Keywords:** anesthetic pharmacology, dexmedetomidine, drug combinations, drug stability, ketamine

## Abstract

Background

Although anesthetic drug admixtures are widely used in clinical practice, the physicochemical stability and photodegradation profile of the S-ketamine-dexmedetomidine (KET-DEX) admixture have not been previously characterized. This study evaluated the physicochemical stability of the KET-DEX admixture under ambient and ultraviolet (UV) light exposure.

Methods

The admixture (0.25 mM) was stored under ambient conditions or exposed to UV light for 24 hours. Physicochemical stability was assessed by high-performance liquid chromatography with diode-array detection (HPLC-DAD), while degradation products were characterized using mass spectrometry. Stability was evaluated based on chromatographic profile, degradation products, and physicochemical parameters.

Results

The KET-DEX admixture remained physicochemically stable for 24 hours under ambient conditions, with no evidence of significant degradation or drug interaction. In contrast, UV exposure induced progressive photodegradation of KET, whereas DEX remained stable.

Conclusions

The KET-DEX admixture demonstrated physicochemical stability under standard laboratory conditions but should be protected from UV light during storage and handling. These findings provide the first evidence of the admixture's stability profile and establish a foundation for future investigations addressing its clinical applicability, efficacy, and safety.

## Introduction

Multimodal anesthesia has become a cornerstone of contemporary anesthetic practice by combining agents with complementary pharmacological mechanisms to improve hypnosis, analgesia, and sedation while reducing dose-dependent adverse effects [1]. Consequently, the preparation of anesthetic drug admixtures in a single solution has become increasingly common in perioperative care, particularly when simplified drug administration or limited availability of infusion pumps favors this approach.

Despite their widespread clinical use, the physicochemical compatibility of many anesthetic admixtures remains insufficiently characterized. Drug incompatibility may result in degradation, precipitation, or the formation of potentially harmful byproducts, potentially compromising efficacy and safety. Therefore, the evaluation of physicochemical stability, degradation products, and potential interactions between combined agents is essential before these formulations can be considered suitable for clinical use. Although the stability of several anesthetic combinations has previously been investigated [2], important knowledge gaps remain, including the physicochemical stability of the S-ketamine and dexmedetomidine (KET-DEX) admixture.

Interest in this combination has increased in recent years because it may provide complementary sedative and analgesic effects while preserving spontaneous ventilation and maintaining hemodynamic stability [3]. Baek et al. evaluated a ketamine-DEX admixture [4]; however, their study included racemic ketamine and lidocaine, preventing conclusions regarding the stability of an admixture containing only the pharmacologically active S-enantiomer (S-ketamine or KET) and DEX.

To our knowledge, no previous study has systematically evaluated the physicochemical stability and photodegradation profile of an admixture composed exclusively of KET-DEX. Therefore, the present study aimed to evaluate the physicochemical stability of this admixture under ambient and ultraviolet (UV) light exposure, providing evidence to support its safe preparation and handling while establishing a foundation for future investigations into its clinical applicability.

## Materials and methods

Experimental design

All analyses and sample handling were performed by certified professionals with expertise in the analytical techniques employed through a collaborative partnership between the Departments of Anesthesiology and Pharmacology. The experimental procedures for physicochemical stability and photodegradation assessment were conducted in accordance with the principles described in the International Council for Harmonisation (ICH) Q1A(R2) [5] and Q1B [6] guidelines for stability testing.

KET and DEX were supplied by Cristália Produtos Químicos Farmacêuticos Ltda. (Itapira, São Paulo, Brazil). The admixture was prepared from commercially available formulations to obtain a final concentration of 0.25 mM for each analyte. Solutions were prepared simultaneously under standardized laboratory conditions (ambient temperature: 25°C) and transferred into clear borosilicate glass vials (final volume: 5 mL). For each experimental condition, three independent preparations were produced and analyzed in triplicate.

The prepared admixtures were subjected to two storage conditions. Under ambient conditions, samples were stored at room temperature under laboratory light and analyzed at 0, one, two, three, four, six, eight, 10, 12, and 24 hours after preparation. For photostability testing, samples were exposed to UV light at 254 nm, following the recommendations of the ICH Q1B guideline (distance from UV source: 10 cm; irradiance: 200 W/m²), and analyzed after 0, one, two, three, four, and 24 hours of exposure.

pH measurement

Before chromatographic analysis, the pH of each admixture was measured using a calibrated digital pH meter. Instrument calibration was performed immediately before analysis using certified standard buffer solutions at pH 4.0 and 10.0 according to the manufacturer's instructions. Whenever calibration values were outside the acceptable limits, the instrument was recalibrated before sample analysis.

High-performance liquid chromatography

Chromatographic analyses were performed using a Shimadzu LC-20AD high-performance liquid chromatography system equipped with a diode-array detector (HPLC-DAD). Separation was achieved using a C18 Ascentis® Express column (2.7 μm, 150 × 4.6 mm) maintained at 40°C.

The mobile phase consisted of water containing 0.1% formic acid (A) and methanol (B), delivered at a flow rate of 0.6 mL/min using the following gradient program: 5-95% B (0-15 min), 95% B (15-20 min), 95-5% B (20-22 min), and 5% B (22-25 min). UV spectra were recorded over the wavelength range of 200-400 nm.

The chromatographic method was validated according to ICH Q2(R2) [7] recommendations with respect to specificity, linearity, precision, and accuracy (or cite the previous validation study if applicable.

Mass spectrometry analysis

Samples exhibiting chromatographic peaks different from those of the parent analytes were further analyzed by tandem mass spectrometry to investigate potential degradation products. Analyses were performed using electrospray ionization (ESI) coupled to an AmaZon SL Ion Trap mass spectrometer (Bruker®, Germany).

Mass spectra were acquired in positive ionization mode over a mass-to-charge ratio (m/z) range of 50-500. Instrument parameters were set as follows: nebulizer pressure of 70 psi, drying gas flow rate of 12 L/min, drying gas temperature of 340°C, capillary voltage of 3.5 kV, and collision energy of 2.5 V for fragmentation experiments.

Stability assessment

Physicochemical stability was assessed by preservation of the chromatographic profile of both parent analytes, maintenance of pH throughout the observation period, and the absence of significant additional chromatographic peaks suggestive of degradation products. A preparation was considered physicochemically stable when at least 90% of the initial analyte concentration was maintained throughout the study period, in accordance with internationally accepted stability criteria. Samples exposed to UV light were additionally evaluated for the formation of degradation products by tandem mass spectrometry.

Data analysis 

Chromatographic peak areas and pH measurements are presented as mean ± standard deviation of triplicate determinations. Changes observed during the study period were evaluated descriptively to determine physicochemical stability under each storage condition.

## Results

Chromatographic stability under ambient conditions

Initially, the HPLC-DAD method was developed to monitor the degradation of each anesthetic, in which the KET and DEX were analyzed separately to determine the peak area of the starting material. Subsequently, we analyzed both substances together, and each peak area from the chromatogram was integrated to compare the mixtures over time and UV light.

**Figure 1:**
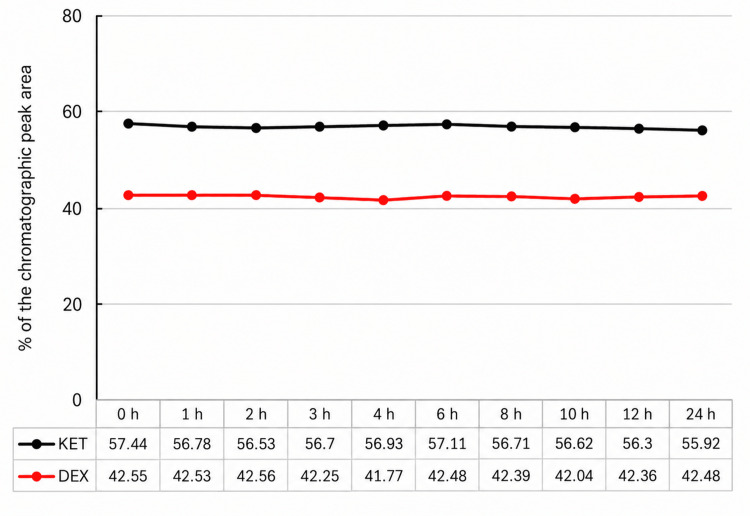
KET-DEX chromatographic peak area over time Variation in chromatographic peak area over time for S-ketamine hydrochloride (KET) and dexmedetomidine hydrochloride (DEX) in the KET-DEX mixture maintained under ambient light and room temperature conditions.

Based on the chromatograms obtained, no reactivity between the mixture components or degradation of the analytes was observed under ambient light and temperature conditions within 24 hours (Figure 1).

**Figure 2:**
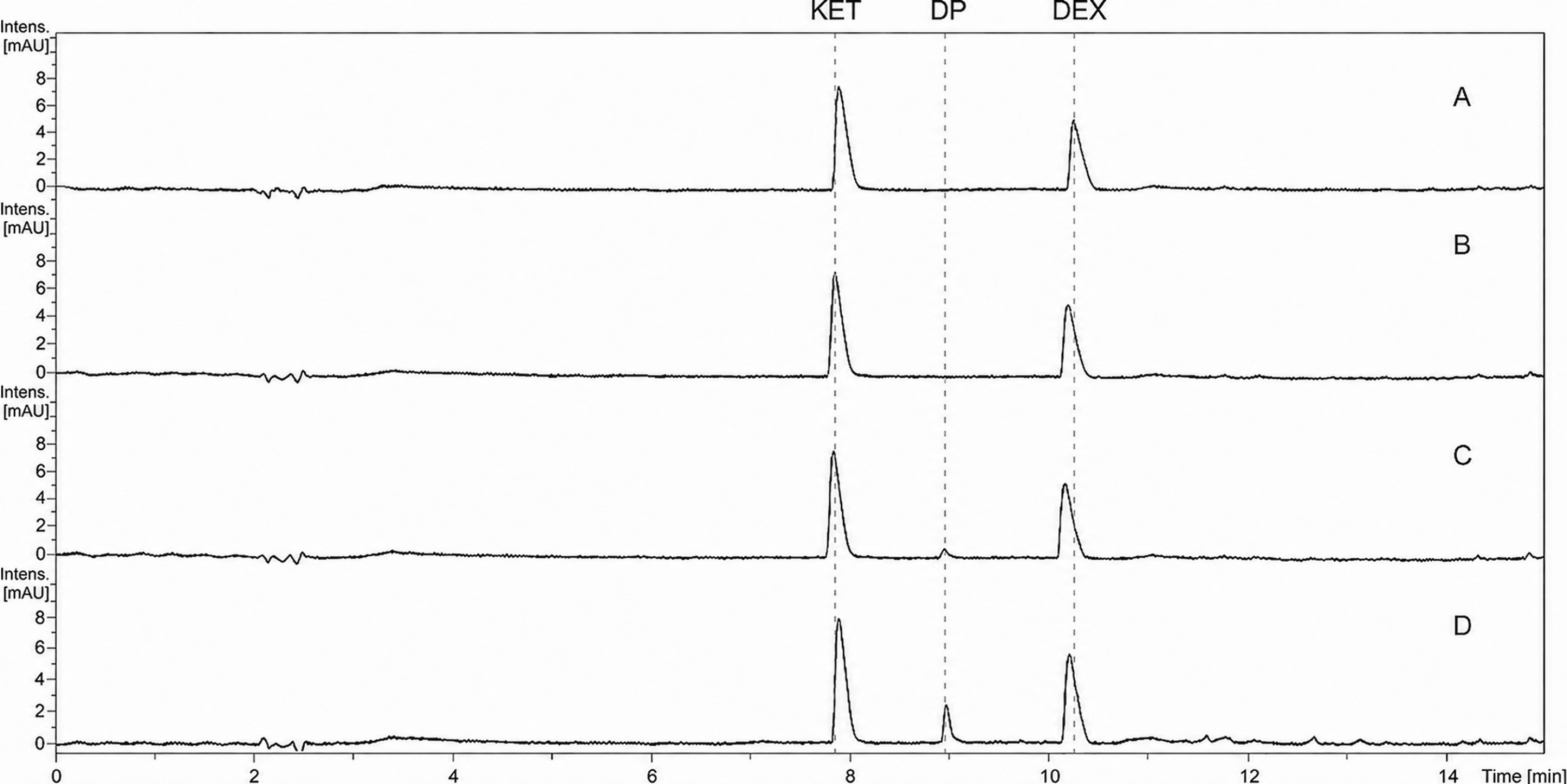
KET-DEX chromatograms Fifteen minutes expansion: chromatograms obtained by high-performance liquid chromatography with diode-array detection (HPLC-DAD) at 254 nm, corresponding to the mixture of s-ketamine hydrochloride (KET), dexmedetomidine hydrochloride (DEX), and the degradation product (DP) under different times and conditions: (A) mixture at time zero; (B) after 24 hours of exposure to ambient light and temperature; (C) after 1 hour of exposure to UV light; (D) after 24 hours of exposure to UV light.

In the data presented in Figure 2, chromatogram A corresponds to the immediate mixture of both anesthetics, and chromatogram B corresponds to the mixture after 24 hours of exposure to ambient light and temperature. In both analyses, only two peaks were observed at retention times of 7.9 minutes and 10.2 minutes, corresponding to KET and DEX, respectively.

UV-induced degradation

**Figure 3:**
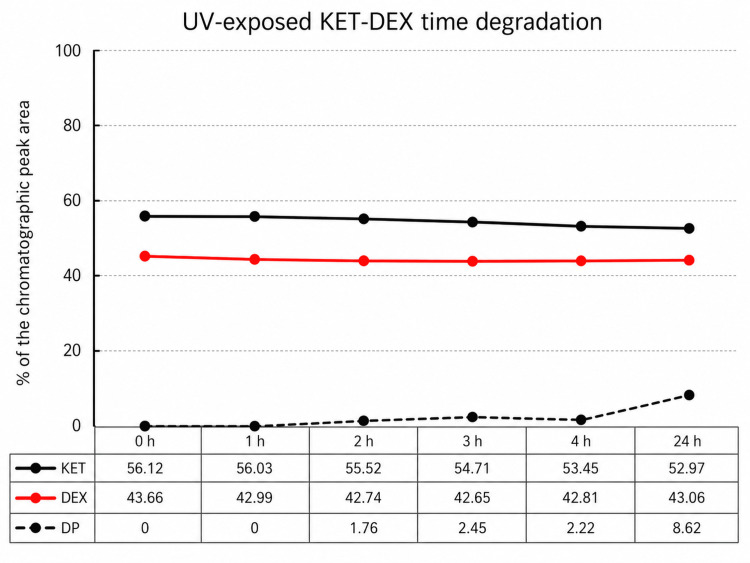
KET-DEX chromatographic peak area under ultraviolet light Variation in chromatographic peak area over time for S-ketamine hydrochloride (KET), dexmedetomidine hydrochloride (DEX), and the degradation product (DP) in the KET-DEX mixture following exposure to ultraviolet (UV) light.

In contrast, chromatograms obtained from samples exposed to UV light showed a degradation product (DP) formation (Figure 3).

**Figure 4:**
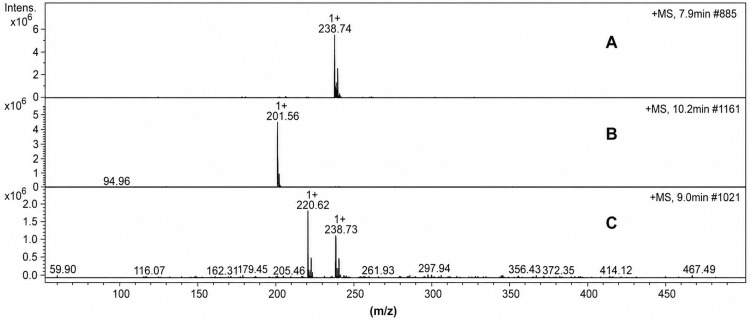
KET-DEX mass spectrometry Mass spectra: (A) S-ketamine hydrochloride (KET; m/z 238); (B) Dexmedetomidine hydrochloride (DEX; m/z 201); (C) Degradation product (DP; m/z 220).

The early appearance and progressive increase of the new peak under UV exposure highlights its time-dependent degradation behavior (Figure 2; chromatograms C and D). The DP was detected at a retention time of nine minutes and analyzed by mass spectrometry (Figure 3) and presented an m/z of 220, an intermediate mass between the two original analytes (KET - m/z 238 and DEX - m/z 201) (Figure 4).

Therefore, the mixtures subjected to stress conditions indicate the formation of a probable degradation product of KET, likely resulting from the loss of a water molecule (m/z 18). However, there is no evidence of any interaction or reactivity between the anesthetics analyzed.

pH stability

**Figure 5:**
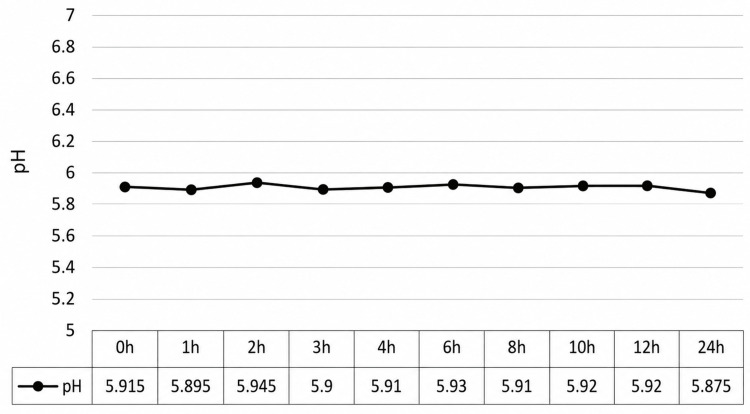
Ambient light pH pH variation over time (hours) in the S-ketamine and dexmedetomidine (KET-DEX) mixture maintained under ambient light and room temperature conditions.

**Figure 6:**
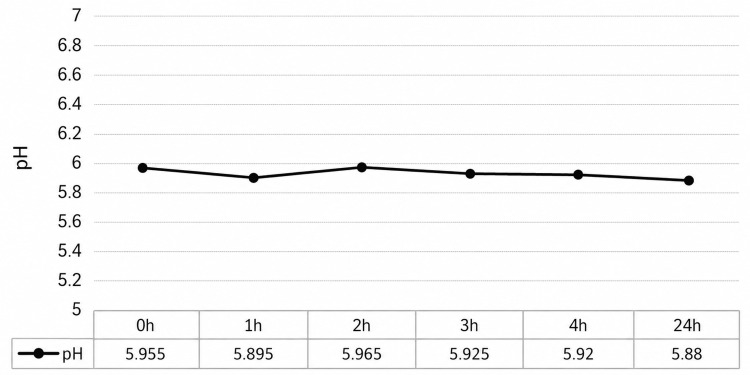
UV-exposed pH pH variation over time (hours) in the S-ketamine and dexmedetomidine (KET-DEX) mixture following exposure to ultraviolet (UV) light.

The pH of the mixtures, both under ambient conditions and under UV light exposure, did not present significative differences within the time intervals and ranged between 5.8 and 5.9 (Figures 5, 6).

## Discussion

The principal finding of this study is that an admixture containing only KET and DEX remained physicochemically stable for 24 hours under standard laboratory conditions, with no evidence of clinically relevant changes in pH, precipitation, turbidity, or chromatographic integrity. In contrast, UV exposure induced progressive photodegradation of KET, whereas DEX remained stable. To our knowledge, this is the first study to systematically characterize both the physicochemical stability and photodegradation profile of an admixture composed exclusively of KET-DEX.

The preparation of anesthetic drug admixtures has become increasingly common in perioperative practice because it simplifies drug administration, reduces the need for multiple infusion pumps and disposable materials, lowers operational costs, and supports the principles of green anesthesia advocated by international anesthesiology societies [1,2,8]. Nevertheless, before these formulations can be safely incorporated into routine clinical practice, their physicochemical compatibility must be demonstrated, since incompatibilities may lead to precipitation, degradation, or the formation of potentially harmful byproducts [2].

Previous studies have reported favorable clinical outcomes with several anesthetic combinations, including propofol-esketamine, fentanyl-droperidol, and lidocaine-sodium bicarbonate admixtures [2-4, 8-13]. However, despite the growing clinical interest in combining KET and DEX for procedural sedation, evidence regarding the physicochemical stability of this admixture has been lacking. Although Baek et al. evaluated a KET-DEX formulation [4], their study involved racemic ketamine combined with lidocaine, while Schenkel et al. investigated a more complex formulation containing KET, DEX, magnesium, and lidocaine [2]. Consequently, neither study established the stability of an admixture composed exclusively of KET-DEX. The present study addresses this important knowledge gap.

The pharmacological rationale for combining these agents further supports the clinical interest in this formulation. KET preserves airway reflexes, spontaneous ventilation, and sympathetic tone, whereas DEX provides sedation and analgesia with minimal respiratory depression, although it may be associated with bradycardia and hypotension [3]. Consequently, their combination may provide complementary sedative and analgesic effects while attenuating some of the adverse effects associated with each drug administered individually. Previous clinical reports have also described satisfactory hemodynamic stability and preservation of spontaneous ventilation using KET-DEX combinations [3,4]. Although the present study did not evaluate clinical efficacy or safety, demonstrating physicochemical compatibility represents an essential prerequisite for future clinical investigations.

The absence of detectable instability under standard laboratory conditions is particularly relevant considering the incompatibilities previously reported for other injectable anesthetic admixtures. Studies have demonstrated micelle formation following lidocaine-propofol admixture, potentially increasing the risk of pulmonary embolism [14,15], precipitation in buffered local anesthetic solutions [16], and alkaline hydrolysis of remifentanil when mixed with propofol, resulting in time-dependent opioid degradation [17]. Our findings suggest that the KET-DEX admixture does not exhibit similar physicochemical incompatibilities under the experimental conditions investigated.

Another important aspect of this study is the exclusive evaluation of S-ketamine (KET) rather than racemic ketamine. Compared with the racemic formulation, the former exhibits greater anesthetic potency and a more favorable pharmacodynamic profile, providing effective analgesia and sedation with potentially fewer adverse effects [18]. This distinction is clinically relevant because KET has become increasingly available in clinical practice, yet no previous study had specifically evaluated the physicochemical stability of an admixture containing only KET and DEX.

The individual analyses of each component further supported the stability profile observed for the combined solution. Similar to the findings reported by Miolo et al. [19], KET demonstrated photodegradation only after UV exposure, with an approximate degradation of 6% and the formation of a degradation product compatible with previously described photodegradation byproducts [20]. Although the biological significance of this compound remains unknown, its identification reinforces the importance of protecting the admixture from ultraviolet light during preparation and storage. DEX remained stable throughout the experimental period, corroborating previous studies demonstrating degradation predominantly under alkaline pH conditions [2,21].

This study has several limitations. First, only physicochemical stability was evaluated, without assessment of pharmacodynamic interactions, pharmacokinetics, clinical efficacy, or patient safety. Second, the experiments were performed under controlled laboratory conditions using a single drug concentration and a 24-hour observation period, which may limit the generalizability of the findings to routine clinical practice. Finally, although UV-induced degradation products were detected, neither their chemical structure nor their potential toxicological significance was investigated. Future studies should evaluate additional drug concentrations, longer storage periods, and real-world storage conditions, as well as determine whether the physicochemical stability demonstrated in this study translates into clinical safety, efficacy, preservation of spontaneous ventilation, and hemodynamic stability through prospective clinical investigations.

## Conclusions

In conclusion, this study demonstrated that an admixture containing only KET and DEX remained physicochemically stable for 24 hours under standard laboratory conditions, with no evidence of clinically relevant changes in pH, precipitation, turbidity, or chromatographic integrity. To our knowledge, this is the first study to systematically characterize both the physicochemical stability and photodegradation profile of this specific admixture.

The observed photodegradation of KET under UV light highlights the importance of appropriate storage and protection from UV exposure during preparation and handling. Although physicochemical stability alone does not establish clinical safety or therapeutic efficacy, it represents an essential prerequisite for the clinical use of multidrug anesthetic admixtures.

These findings provide a scientific foundation for the safe preparation and handling of the KET-DEX admixture and support future studies investigating its long-term stability, additional concentrations, and clinical performance in patients, particularly in settings where preservation of spontaneous ventilation and hemodynamic stability is desirable.
